# Disease networks identify specific conditions and pleiotropy influencing multimorbidity in the general population

**DOI:** 10.1038/s41598-018-34361-3

**Published:** 2018-10-29

**Authors:** A. Amell, A. Roso-Llorach, L. Palomero, D. Cuadras, I. Galván-Femenía, J. Serra-Musach, F. Comellas, R. de Cid, M. A. Pujana, C. Violán

**Affiliations:** 1 Department of Mathematics, Technical University of Catalonia, Castelldefels, Barcelona, 08860 Catalonia Spain; 2 Jordi Gol University Institute for Research Primary Healthcare (IDIAP Jordi Gol), Barcelona, 08007 Catalonia Spain; 3 Autonomous University of Barcelona, Bellaterra, 08193 Catalonia Spain; 4 ProCURE, Catalan Institute of Oncology (ICO), Oncobell, Bellvitge Institute for Biomedical Research (IDIBELL), L’Hospitalet del Llobregat, Barcelona, 08908 Catalonia Spain; 5 Statistics Department, Foundation Sant Joan de Déu, Esplugues, 08950 Catalonia Spain; 6 GCAT-Genomes for Life, Germans Trias i Pujol Health Sciences Research Institute (IGTP), Program for Predictive and Personalized Medicine of Cancer (IMPPC), Badalona, 08916 Catalonia Spain

## Abstract

Multimorbidity is an emerging topic in public health policy because of its increasing prevalence and socio-economic impact. However, the age- and gender-dependent trends of disease associations at fine resolution, and the underlying genetic factors, remain incompletely understood. Here, by analyzing disease networks from electronic medical records of primary health care, we identify key conditions and shared genetic factors influencing multimorbidity. Three types of diseases are outlined: “central”, which include chronic and non-chronic conditions, have higher cumulative risks of disease associations; “community roots” have lower cumulative risks, but inform on continuing clustered disease associations with age; and “seeds of bursts”, which most are chronic, reveal outbreaks of disease associations leading to multimorbidity. The diseases with a major impact on multimorbidity are caused by genes that occupy central positions in the network of human disease genes. Alteration of lipid metabolism connects breast cancer, diabetic neuropathy and nutritional anemia. Evaluation of key disease associations by a genome-wide association study identifies shared genetic factors and further supports causal commonalities between nervous system diseases and nutritional anemias. This study also reveals many shared genetic signals with other diseases. Collectively, our results depict novel population-based multimorbidity patterns, identify key diseases within them, and highlight pleiotropy influencing multimorbidity.

## Introduction

Multimorbidity, defined as the co-occurrence of two or more diseases in a given individual, poses a major challenge to quality of care, and emerges as an important issue when considering activity and effort in health systems^1,2^. Multimorbidity is commonly associated with chronic conditions, but non-chronic or acute diagnoses, such as those related to falls, also contribute to its occurrence^3^. Chronic diseases are particularly relevant because of their rising prevalence and burden in aging societies, where they incur substantial costs to health care systems. In fact, the economic cost per multimorbid patient is 3–5 times that of non-multimorbid cases^4,5^. As highlighted by the World Health Organization, chronic diseases have reached epidemic proportions and constitute the leading causes of death in the world^6^. In Europe, an estimated 50 million people —approximately 7% of the total population— suffer from multimorbidity^7^. This percentage is even higher (>55%) among the elderly^8^. Even so, health systems do not meet the needs of multimorbid patients; the structures are typically “disease oriented” and “non-integrative”. Thus, care is generally organized around specific medical specialties, an approach that leads to fragmentation, which, in turn, may lead to over-prescription, over-hospitalization, and poor patient satisfaction^9,10^. Therefore, there is a clear need to improve care for individuals with multimorbidities, but this requires a much more detailed understanding of the trends of disease associations than we currently possess. In addition, there is a need to identify genetic factors influencing multimorbidities, which might then constitute new tools for clinical prevention and monitoring.

To date, the study of age- and gender-dependent disease associations at the population level has mainly focused on chronic^1,11^ and/or specific^12,13^ conditions. Broader disease analyses have been performed, but have centered on high-order classifications^14^, the elderly^15^, and/or relatively small cohorts^2^. Network-based approaches have the potential to uncover unexpected relationships between diseases^14–22^. To apply these approaches, systematic and detailed high-quality clinical annotations of a large number of individuals are required. In parallel, collection and analysis of biological samples in the same population can provide the means to identify shared genetic factors among diseases linked to multimorbidity^23,24^. Here, by constructing and analyzing disease networks from high-quality primary health care data, and by integrating the results with genome-wide association studies (GWASs) of individuals from the same population, we identify key diseases, their cumulative risk trends and genetic factors influencing multimorbidity.

## Results

### Disease networks built from primary health care data

**Figure 1:**
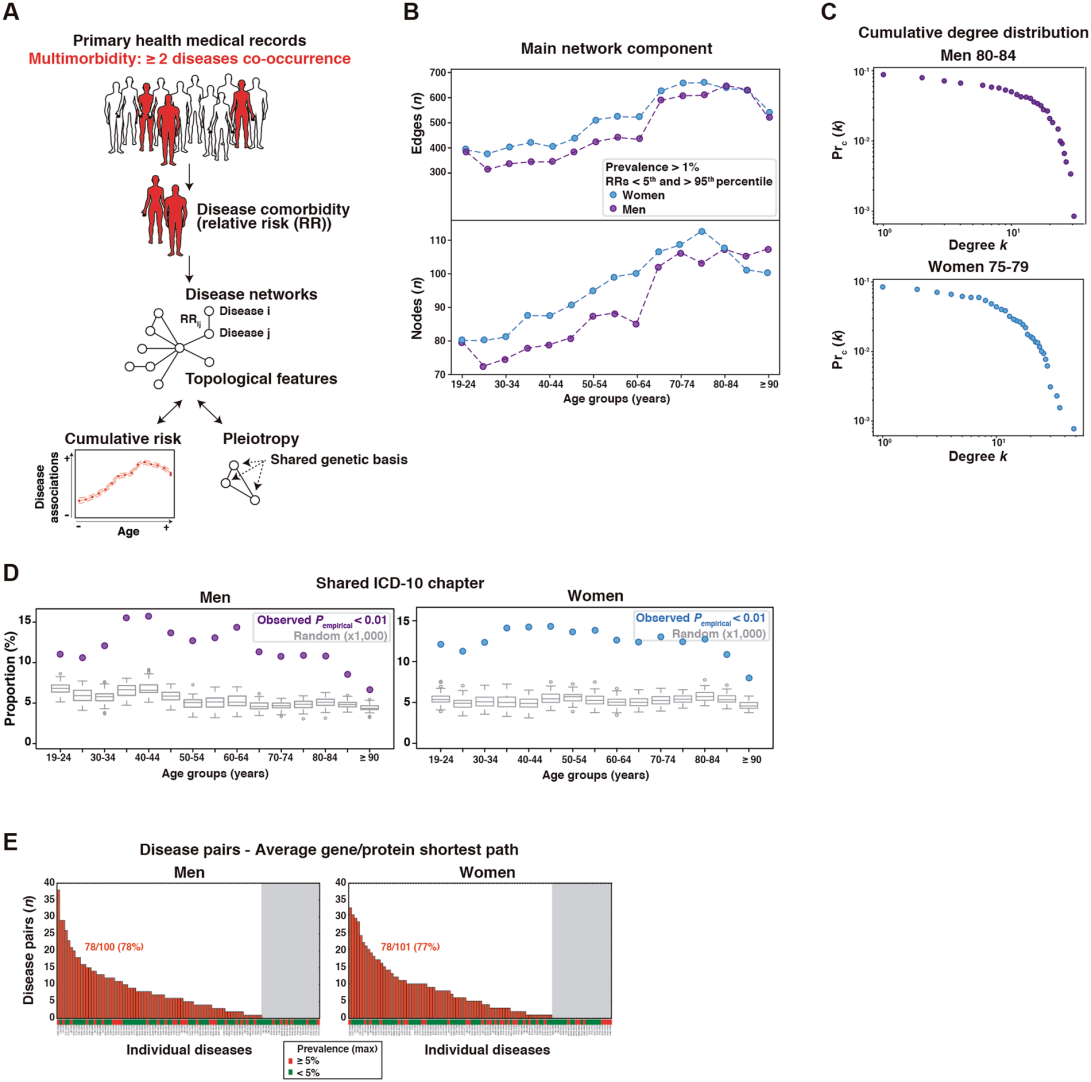
Study design and disease networks. (**A**) Strategy for the identification of diseases and genetic factors influencing multimorbidity. Network nodes and edges correspond to diseases and relative risks (RRs), respectively, and were constructed using primary health records from the Catalan general population. The human figures were created by Freepik. (**B**) Distributions of the number of nodes and edges in each main network component across strata and by gender. (**C**) Exponential decay of cumulative degree (*k*) distributions of two example disease networks as depicted. (**D**) Proportions of linked ICD-10 codes that share a clinical chapter; box-plots show the results of 1,000 permutations and the observed value for each stratum network is indicated by a dot. (**E**) Number of diseases with causal genes/proteins included in the molecular network that revealed at least one disease association with a smaller shortest path than expected at random. The ordered bars indicate the number of disease associations that match this criterion for each disease (ICD-10 codes are indicated on the x-axis). The gray zone indicates diseases that do not match the criterion. A prevalence threshold is also depicted.

A dataset from the electronic primary health care records of Catalonia, a Mediterranean region with more than seven million individuals, was analyzed for age- and gender-centered disease network topological properties that may be associated with multimorbidity and/or pleiotropy (Fig. 1A). This primary health dataset, known as SIDIAP-Q, comprises records from the universal coverage health care system and high-quality clinical annotations based on validated scores^25,26^. Patient diagnoses were based on the International Statistical Classification of Diseases and Related Health Problems, 10^th^ revision (ICD-10)^27^. A total of 1,749,722 individuals (23.5% of the Catalan population) aged at least 19 years and with two or more open recorded diagnoses between 1^st^ January and 31^st^ December 2010 were grouped by 5-year intervals or strata (from 19–24 to ≥90 years old) and by gender, and included in this study (Supplementary Fig. S1a). To investigate the impact of diseases and multimorbidities that are most relevant to the general population, we only considered diagnoses with a prevalence of ≥1% (Supplementary Table S1) and that were associated with any other disease by a measure of comorbidity strength (hereafter relative risk (RR)^15,28^) included in the bottom or top five percentiles across the 15 age strata of men and women. These thresholds corresponded to RR estimates of <0.8, which suggests mutually exclusive diseases, or >1.6, which suggests co-occurring or comorbid diseases, respectively, across all the strata (Supplementary Fig. S1b and Supplementary Table S2). The RR estimates were positively correlated (Spearman’s correlation coefficients (*ρ*) = 0.82–0.88, *P* < 10^−16^) with the Jaccard index, a statistic frequently used to measure the similarity of sample sets. However, this index is not appropriate for relatively rare observations and cannot distinguish between different directions of association^29^.

For each stratum, a network of morbidities was derived in which nodes represent diseases and edges represent RRs. The main network components included more than 70 nodes or nosological entities, and 300 edges or disease associations (Fig. 1B). Except for the elderly, these components were found to be bigger in women, which is consistent with a higher prevalence of female multimorbidity^2,10^. The cumulative distributions of the number of edges by nodes (degree (*k*) distribution) revealed exponential decays (Fig. 1C). This is a similar pattern to that of mortality following emergency medical admission^30^ and is inversely related to epidemic spread^31^. In addition, all observed morbidity networks exhibited a predictable property of ‘small-world-ness’^32^ (Supplementary Fig. S2), by which most nodes or diseases can be reached from every other node through a relatively small number of edges^33^. Therefore, the constructed disease networks are coherent with previous knowledge and reveal expected systems-level features.

### Clinical coherence of the disease networks

To assess the clinical coherence of the networks, we performed 1,000 permutations of the associated (based on RRs) ICD-10 codes in each stratum and computed the proportion of code pairs sharing a higher-level clinical classification or chapter; there were 21 of these^27^. In all strata and for both genders, none of the random sets showed a higher proportion of shared clinical chapters than that of the real networks (Fig. 1D). Next, the clinical coherence of the networks was evaluated using the functional and molecular interactions of the underlying genes and/or proteins (genes/proteins). The ICD-10 codes were linked to the genes/proteins associated to each condition based on the phenotype-genetic associations from the Online Mendelian Inheritance in Man (OMIM)^34^. We hypothesized that coherent disease associations frequently show relatively small shortest interaction paths between the underlying genes/proteins. Thus, approximately 78% of the diseases with an OMIM annotated gene/protein included in a molecular network showed at least one disease association with a smaller shortest path than randomly expected, and there was no bias with respect to prevalence differences (Fig. 1E). Therefore, the disease networks are also coherent based on higher order clinical annotations and phenotype-genetic associations.

### Identification of central diseases

**Figure 2:**
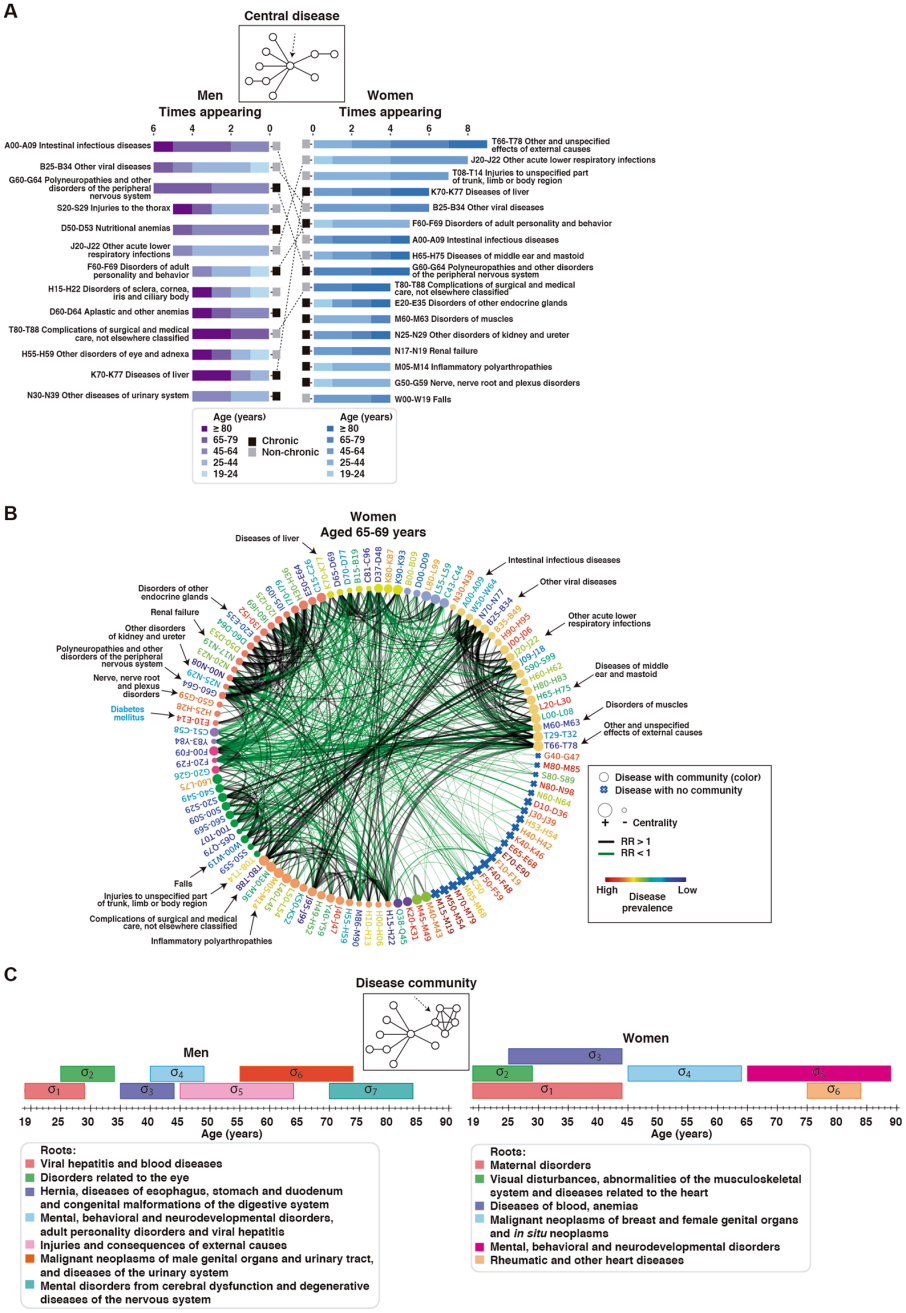
Central diseases and network communities. (**A**) Diseases emerging as topologically central in men and/or women. The number of appearances (in different strata), the corresponding ages, and the specific condition (chronic or non-chronic) are shown. The dotted lines indicate diseases found to be common to men and women. (**B**) Disease network for women aged 65–69 years and depicting diseases (ICD-10 codes) identified as central in this gender. The node corresponding to “Diabetes mellitus” (not central) is also indicated (blue font). The node sizes reflect centrality value and their colors indicate communities. Edge thickness is proportional to the magnitude of the RR estimation; black indicates RR > 1.6 and green indicates RR < 0.8. Disease prevalence is shown by font colors as indicated in the inset. (**C**) Network communities appearing in at least two consecutive strata in men or women. The disease roots of each community are depicted in the insets.

Having established their coherence, we analyzed the networks in order to detect diseases with a major impact on multimorbidity. A modified version of the PageRank^35^ algorithm was applied to take into account the edge weights indicated by the RRs (see Methods). Thus, 13 and 17 diseases appeared at least four times among the 10 most central diseases across the strata in men and women, respectively (Fig. 2A). Seven diseases (including chronic and non-chronic conditions) were common to both genders and comprised critical diagnoses across different ages, such as “Disorders of adult personality and behavior” (Fig. 2A). Non-chronic, acute conditions, such as injuries and infections, also proved to be central in several strata, building on previous observations in older patients^3^.

Central nodes commonly show multiple edges linking different “disease communities” (subsequent section). Diseases that are highly prevalent in the population, like “Diabetes mellitus”, also have a relatively large number of edges, but these are mainly linked to diseases in the same community (Fig. 2b). Nonetheless, consistent with epidemiological observations^36^, the strongest association with “Diabetes mellitus” corresponded to “Polyneuropathies and other disorders of the peripheral nervous system” (RR = 3.73, *P* < 10^−16^), and this condition emerged as central in this study (Fig. 2A,B). According to their topological feature, deletion of central nodes led to a higher number of network components than that of randomly expected in 3/15 and 12/15 of the male and female disease networks with edges of RRs > 1, respectively. Conversely, no such impacts were observed when central nodes were deleted in networks with edges of RRs < 1 (Supplementary Fig. S3). Collectively, the above data identify chronic and non-chronic conditions with a potential major role in multimorbidity.

### Main disease communities

To analyze the patterns of disease aggregations, densely connected sets of nodes or network communities appearing in at least two consecutive strata were identified. The diseases commonly present across the strata comprised the “roots” of the communities. Thus, recognized temporal patterns associated with gender-specific diseases were observed: for instance, cancer-associated communities were identified spanning the 45–64- and 55–74-year-old groups for women and men, respectively (Fig. 2C and Supplementary Table S3). This analysis also highlighted disease communities that may require further health-care efforts based on their sustained presence over time, in particular, a community with root “Injuries and consequences of external causes” in men aged 45–64 years, and a community with root “Mental, behavioral and neurodevelopmental disorders” in women aged 65–89 years (Fig. 2C). Therefore, community-aggregated diseases identify specific multimorbidity patterns, providing a means for following up clustered associations with age.

### Unexpected bursts of disease associations leading to multimorbidity

**Figure 3:**
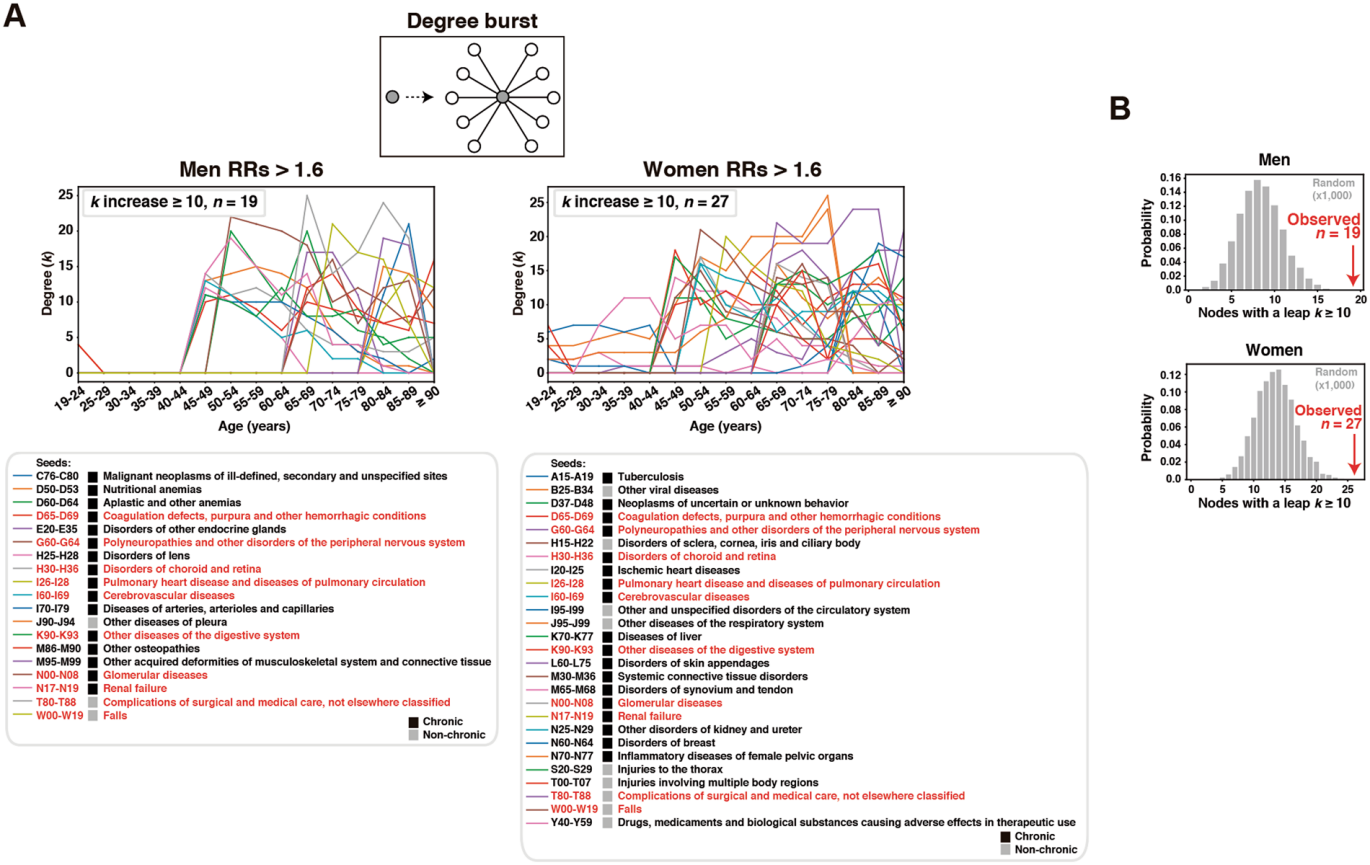
Multimorbidity bursts. (**A**) Age-based trajectories of nodes with large degree leaps; ≥10 edges (RRs > 1.6) over time. The left and right panels show results for men and women, respectively. The corresponding diseases are listed below each graph, and their chronic or non-chronic status is also shown. (**B**) Distribution of connectivity leaps in 1,000 random networks with the same degree distribution and connectedness as that of the real morbidity networks with RRs > 1.6. The y- and x-axes depict the probability and number of nodes with leaps of ≥10 edges, respectively; red arrows indicate the values observed in the real networks.

The progression of cumulative disease associations was further analyzed at the level of node degrees. The number of edges (considering only RRs > 1.6) per node was computed across all strata, and nodes with relatively large leaps in their degree (*k*) were identified; i.e., representing a large increase in the number of associations for a given disease, from a younger to an older stratum. This analysis revealed 19 and 27 nodes in men and women with leaps of *k* ≥ 10, respectively, and these included 10 diseases common to the two genders (Fig. 3A and Supplementary Table S4). To assess the significance of these multimorbidity bursts, the results were compared with those of 1,000 equivalent random networks in each stratum and gender, preserving the degree distribution and connectedness of each corresponding real network. Remarkably, none of the random networks showed a distribution with a greater or equal number of large-degree leaps than the real networks (one-sided *P*_empirical_ < 0.001; Fig. 3B). Four and seven of the 19 and 27 aforementioned diseases, respectively, were previously classified as central, and two were present in both genders: “Complications of surgical and medical care, not elsewhere classified” and “Polyneuropathies and other disorders of the peripheral nervous system” (Figs 2A and 3A). Therefore, particular diseases, some of which also play a central role in networks, act as seeds for multimorbidity.

Most of the bursts (72% (26/36) in men and women) corresponded to chronic conditions acting as seeds (Fig. 3A and Supplementary Table S4). However, the non-chronic diagnoses “Complications of surgical and medical care, not elsewhere classified” and “Falls” also emerged in this analysis in both genders (Fig. 3A). The former condition suggests that prevention of multimorbidity in primary health care should take into account surgical interventions in hospitals. In addition, the identification of “Falls” is consistent with the findings of recent epidemiological studies in the elderly^37,38^, so monitoring these acute conditions could further improve the management of multimorbidity bursts, particularly in middle-aged women, as suggested by our study (Fig. 2A).

### Trajectories of cumulative risks

**Figure 4:**
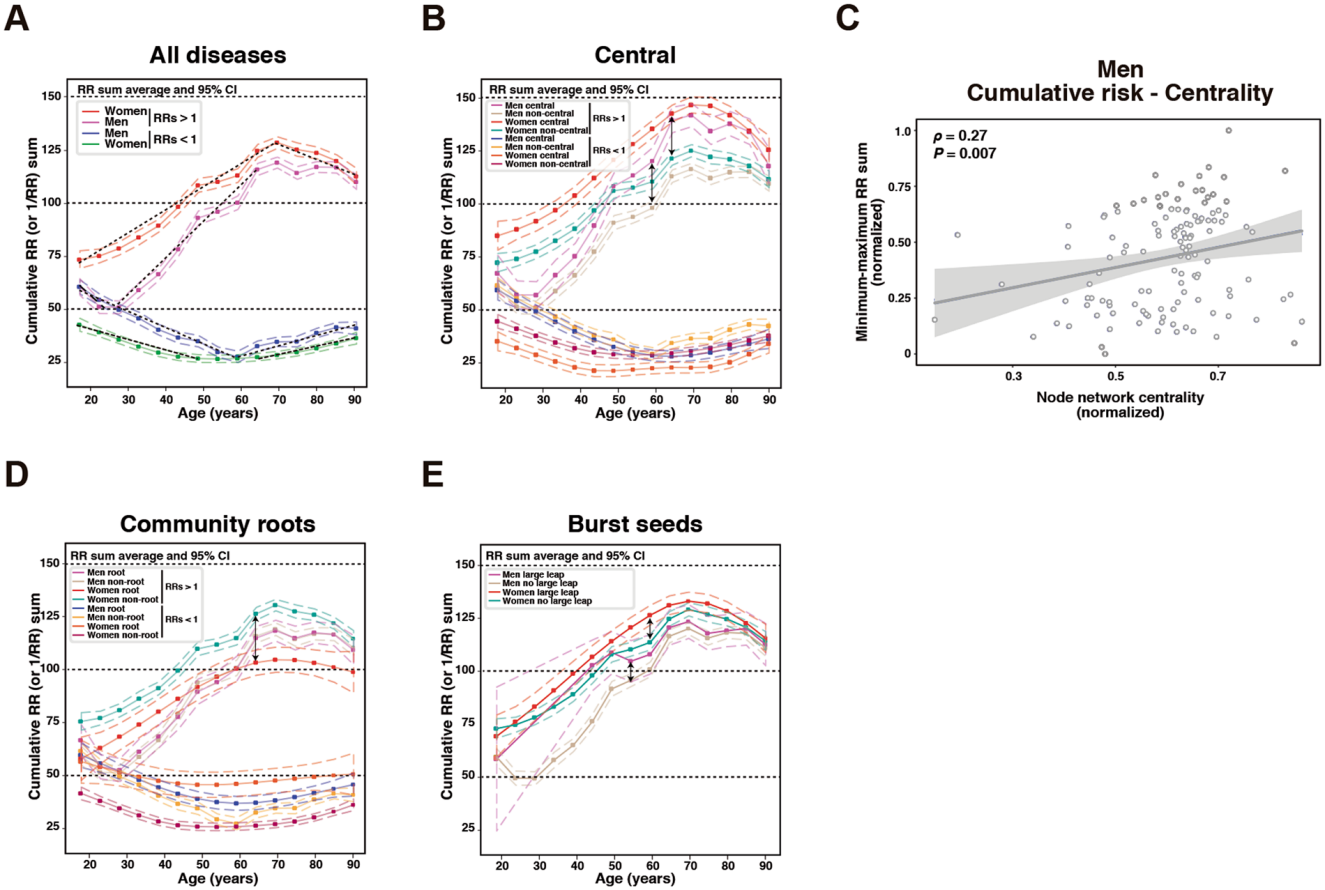
Cumulative risk trends. (**A**) Average and 95% CI of RR sums by gender and age group. The dotted lines indicate slopes significantly different from zero. (**B**) Average and 95% CI of RR sums of diseases identified as central in the networks or as other, non-central diseases. The arrows indicate the cumulative risk differences between central and non-central diseases in men (60 years) and women (65 years). (**C**) Graph showing the correlation between the average centrality value of each node across all networks in men, and the difference between the minimum and maximum RR sums of each disease. The linear trend and 95% CI (shaded area) are shown. (**D**) Average and 95% CI of RR sums of diseases identified as network community roots or other diseases (i.e., non-roots). The arrow indicates the cumulative risk difference between non-root and root diseases in women (65 years). (**E**) Average and 95% CI of RR sums of diseases identified as having large degree leaps (≥10 edges, and excluding those that are also central) or other diseases. The arrows indicate cumulative risk differences between disease sets with large leaps and no large leaps in men (55 years) and women (60 years).

The results above have shown unexpected bursts of disease associations that may have an important role in the emergence of multimorbidity. However, it remains unknown if there are differential trends of cumulative disease associations among the different types of network nodes. The progressive aggregation of diseases was evaluated by analyzing the trajectories of the sum of all RRs for each disease as a function of age. This analysis was independent of the initially defined RR thresholds and considered all diseases with ≥1% prevalence. While the sum of RRs < 1 (using their inverse value, 1/RR) revealed mostly flat or smoothly decreasing profiles in both genders, substantial increasing trends were observed for summed RRs > 1 (Supplementary Fig. S4). To assess differences in the trends, the 95% confidence interval (CI) estimates of each RR sum distribution were computed; thus, the trends for women and men did not overlap for most of the age groups (Fig. 4A). Women had higher average RR sums, but men, particularly those aged 30–64 years, had a steeper slope (Fig. 4A). A coincidence test indicated that all four distributions (by gender and/or effect) were significantly different (*P* ≤ 0.001). Remarkably, the global increase of summed RRs > 1 was found to be approximately 60% and 40% in men and women, respectively, further highlighting the relevance of multimorbidity.

Next, the trends of the disease sets classified above as central, community roots, or with large degree leaps were analyzed. Consistent with their key role in multimorbidity progression, the central diseases in men and women showed higher RR sums than all other diseases (*P*_*coincidence*_ ≤ 0.002; Fig. 4B). Again, women had higher sums, but the slopes were steeper in men (Fig. 4B). Building on these observations, analysis of the global correlation between the average centrality of each node across all the networks, and the difference between the minimum and maximum RR sum of each disease across all strata, revealed a positive association in men (*ρ* = 0.27, *P* = 0.007; Fig. 4C). Therefore, node centrality in male disease networks is linked to its relative importance in accumulating disease associations with age. The equivalent analysis in female networks did not reveal a significant association, possibly due to the lower minimum-maximum cumulative risk difference (Fig. 4A).

Subsequently, opposite of what was seen for the central diseases, but consistent with the network topology, the diseases identified above as community roots had lower RR sums than did all other diseases, particularly in women (*P*_*coincidence*_ < 0.001 relative to central; Fig. 4D). However, diseases that are seeds for multimorbidity bursts (excluding those that are also central) also had a higher cumulative risk of comorbidities (*P*_*coincidence*_ ≤ 0.002 relative to roots; Fig. 4E). These results were corroborated using the cumulative average of RRs for each disease (Supplementary Fig. S5). Therefore, network-based features identify different types of diseases relative to their cumulative risk leading to multimorbidity.

### Centrality and pleiotropy linked to causal genes

**Figure 5:**
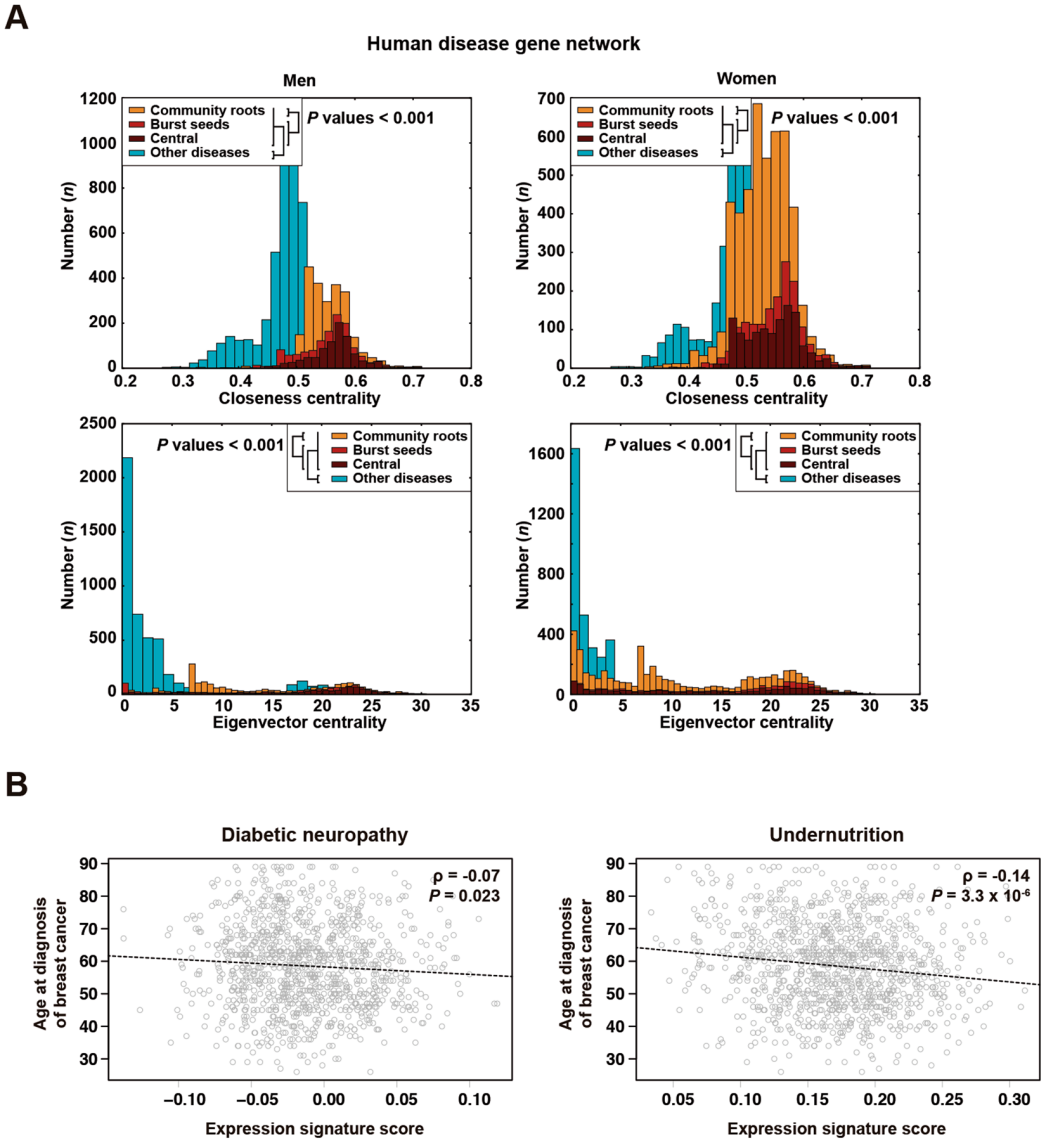
Centrality and pleiotropy linked to causal genes. (**A**) Graphs showing the distributions of closeness and eigenvector centrality measures for different types of causal genes as indicated in the insets. The results correspond to the analysis of the curated human disease gene network and are shown for men and women disease sets derived from the SIDIAP-Q networks study. The Wilcoxon test *P* values of the comparisons of distributions are shown. (**B**) Scatter plots depicting the negative correlations between the gene expression signatures (all genes included) that define diabetic neuropathy (left panel) or undernutrition (right panel) and age at diagnosis of breast cancer. The stage-adjusted linear regression coefficients and their corresponding *P* values are shown.

The identified diseases underlying multimorbidity —particularly those linked to network centrality and/or bursts of disease associations— may be caused by genes that, as a consequence, influence multiple human disorders. To test this hypothesis, we analyzed a curated human disease gene network in which two genes are connected if they are causative of the same disease^39^. Using this independent dataset and two different measures of network centrality, the causal genes of the central and burst-seed diseases were found to be more central than that of the community-root diseases in both genders (Mann-Whitney *P* values < 0.001; Fig. 5A). Intriguingly, the causal genes of community-root diseases were also found to be more central than that of the rest of diseases (Mann-Whitney *P* values < 0.001; Fig. 5A), which further highlights the link of these conditions with major disease aggregations through age (Fig. 2C). Topological analyses of the corresponding gene products in a curated interactome network^40^ also revealed that all three sets (i.e, central, burst-seed, and community-root) have higher centrality than that of other gene products (Supplementary Fig. S6). In contrast to the gene network results, there were not centrality differences between community-root and central or burst-seed sets using men data, and differences were only marginally significant using women data (Supplementary Fig. S6). This observation might denote a non-lineal relationship between genetic causality and diversity of protein function.

As indicated above, one condition emerged as relevant in both the centrality and burst analyses: “Polyneuropathies and other disorders of the peripheral nervous system”. This condition had fewer recognized associations with “Malignant neoplasms, stated or presumed to be primary” and “Nutritional anemias” in women (Supplementary Table S4). Following on these observations, the concordance of gene expression alterations underlying the three diseases was assessed^41–43^. Higher overlaps than expected by chance were observed between the gene expression signatures from the three diseases (χ^2^ *P* < 0.002). Genes involved in lipid metabolism were found to be common to all three diseases (Supplementary Table S5). By contrast, no significant overlap was found when compared to differentially expressed genes in lung adenocarcinomas^44^. Furthermore, the expression scores for the signatures characteristic of undernutrition^42^ and diabetic neuropathy^43^ were found to be negatively correlated with age at diagnosis of breast cancer (Fig. 5B). Therefore, the central and burst-seed diseases are caused by genes that in turn play a central role in the human disease gene network, and we provide evidence of shared gene expression alterations between diabetic neuropathy and undernutrition that promote breast cancer.

### Shared genetic factors among diseases linked to multimorbidity

**Figure 6:**
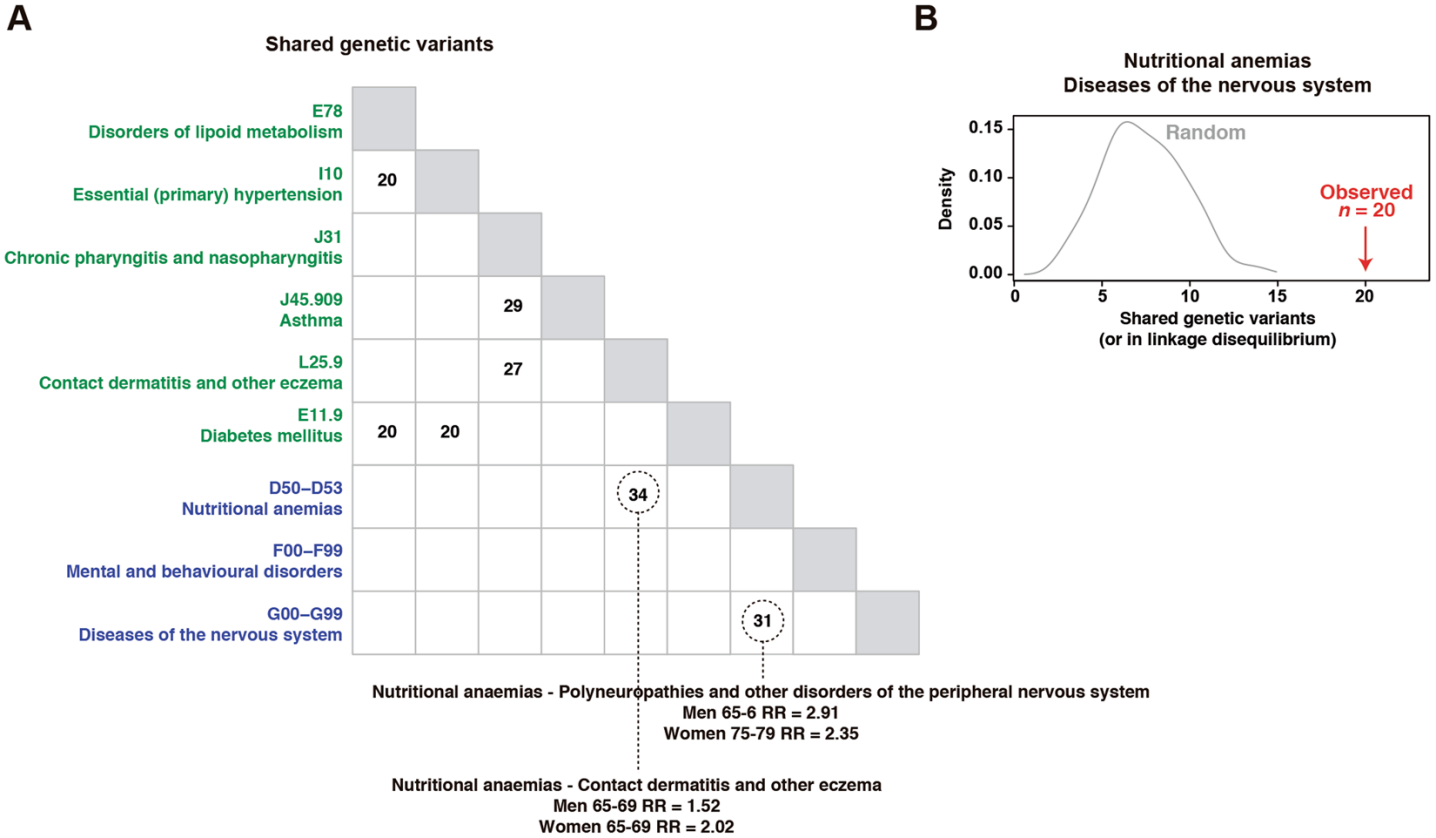
Shared genetic factors among diseases linked to multimorbidity. (**A**) Matrix depicting pairs of central (blue) and common (green) diseases, and instances with a significant number of shared genetic variants relative to random GWASs (numbers of variants are shown). The corresponding RRs are shown for instances linking central diseases. (**B**) Distribution of shared genetic variants (also considering those in linkage disequilibrium) among 100 random sets of 31 variants and observed value of shared signals between “Nutritional anemias” and “Diseases of the nervous system”. The y- and x-axes depict the probability and number of shared variants, respectively; red arrows indicate the value observed.

To further evaluate disease associations at the level of shared genetic factors, a GWAS was performed in the same population as the SIDIAP-Q disease networks study (Genomes for Life)^45^. This investigation focused on central diseases with detailed clinical definitions and on common diagnoses with more than 200 cases included in the cohort (three and nine diseases, respectively; Supplementary Table S6). The application of a genome-wide association pairwise approach^46^ revealed that central diseases tended to share, on average, a greater number of significantly associated variants than the nine common diseases (20 *vs*. 11 significant signals). Subsequently, seven of the 36 possible non-redundant disease pairs showed a higher number of shared variants than that of 100 random GWASs (Fig. 6A). Importantly, these seven pairs corresponded to RRs > 1.5 (*P* < 10^−3^) across at least two strata in both genders, which reinforces their epidemiological relevance.

Besides expected overlaps (e.g., shared signals between “Diabetes mellitus” and “Disorders of lipoid metabolism” or “Essential (primary) hypertension”), there were shared genetic associations between “Nutritional anemias” and “Diseases of the nervous system” (Fig. 6A), which includes polyneuropathies. Thirty-one significant association signals were detected in this comparison and, notably, three of them were also found to be in linkage disequilibrium (LD, *D*’ > 0.99) with variants previously identified as influencing multiple human traits^46,47^ (Supplementary Table S7). Most importantly, 17 of the 28 remaining shared signals were found to be in linkage disequilibrium with GWAS results involving one or more other human disorders or traits (Supplementary Table S7). This proportion of 20/31 shared signals was found to be higher than the average proportion of 100 sets of 31 randomly chosen genetic variants (*P*_*empirical*_ ≤ 0.01; Fig. 6B). The neighbor genes of these pleiotropic signals were found to be significantly enriched (false discovery rate (FDR)-adjusted *P* = 2.1 × 10^−7^) in loci linked to smoking cessation versus dependence^48^. Intriguingly, smoking is an established lifestyle factor associated with multimorbidity^49,50^. Therefore, key disease associations linked to multimorbidity are influenced by shared genetic factors, which in turn may be associated with important lifestyle factors.

None of the 31 signals appeared to be an expression quantitative trait locus (eQTL) when exploring the GTEx database (v6.0)^51^. However, when variants in LD were considered, the expression of 17 genes may be associated (Supplementary Table S8). Notably, seven of these genes were found to be altered in thyroid tissue, which represents a higher enrichment than expected by chance (χ^2^ *P* = 9 × 10^−6^). This observation might be in concordance with observational studies in animals and humans linking impaired thyroid metabolism to iron-deficiency anemia^52^. In addition, thyroid deregulation (underactive thyroid) is a risk factor for peripheral neuropathy, which overall provides a tissue-based mechanistic hypothesis for the observed multimorbidity and pleiotropy.

## Discussion

Our results show that specific topological features of disease networks identify conditions with a key role in the emergence and/or progression of multimorbidity in the general population. The causal genes of these key conditions also occupy central positions in the network of human disease genes, which is consistent with their predicted pleiotropic effects. In addition, this study reveals shared genetic factors among diseases linked to certain multimorbidities and, in particular, highlights associations between breast cancer, diabetic neuropathy, and nutritional anemia, and between diseases of the nervous system and nutritional anemias.

Three types of diseases are identified in this study: central, which include chronic and non-chronic conditions, accumulate relatively higher risk of multimorbidity with age in both genders; “community roots”, which accumulate less risk, but indicate major disease aggregations with age; and “burst seeds”, which nucleate diagnoses for 10 or more conditions in a single individual. In the biomedical scenario, central diseases may be interpreted as those more likely lead to multimorbidity or more likely appear in a given multimorbid patient. In an analogous manner, their causal genes have the potential to influence multiple diseases and, therefore, they may be functionally linked to different molecular process and/or signaling pathways^53–55^. A particular type of central disease with a key role in multimorbidity corresponds to those identified as “burst seeds”, which show a sharp accumulation of disease associations. The causal genes of this type of diseases may also harbor pleiotropic effects, but one can speculate that other biological, environmental and/or lifestyle factors critically contribute to the observed burst effect. Finally, the function of causal genes for “community root” diseases may be more specific at the molecular, cellular and/or tissue level.

The observed multimorbidity bursts are generally linked to chronic diseases and, thus, clinical studies of identified seed conditions may be able to improve prevention strategies and health care policies^9,10^. Nonetheless, two acute conditions (“Complications of surgical and medical care, not elsewhere classified” and “Falls”) also emerge as central and mediating bursts, so their integration in prevention could further help improve multimorbidity care, and not only in the elderly^37,38^. However, there are significant differences in the cumulative risk trends between men and women, which therefore should also be taken into account when preventing and/or managing multimorbidity. “Polyneuropathies and other disorders of the peripheral nervous system” and, again, “Complications of surgical and medical care, not elsewhere classified” appear to be particularly relevant in both genders. The identification of the latter is additional evidence that attention to multimorbidity in primary care should be coordinated with programmed activities in secondary and tertiary care^3,56^. In contrast to central diseases, network communities provide evidence to detect clustered aggregations across sequential age groups. Thus, community roots should not be the focus of cumulative risk analyses, but they can potentially assist in identifying the most frequent disease aggregations.

Monitoring of individuals diagnosed with diseases identified in this study, in combination with analyses of pleiotropic factors, could potentially reduce the current impact of multimorbidity on health care systems. Crucially, our study shows that the causal genes of central and burst-seed diseases occupy a central position in a genetic network of human disorders, which further endorses their relevance in multimorbidity. Therefore, analyses of these causal genes may be useful for monitoring and/or predicting multimorbidity. Specifically, lipid metabolism appears to be commonly perturbed in breast cancer, diabetic neuropathy, and nutritional alterations, which is also consistent with the proposed causal links between cancer, diabetes, and obesity^57^. At the germline level, our GWAS in individuals of the same population in which disease networks are studied has identified seven pairs of diseases with a significant number of shared genetic factors. These pairs include “Nutritional anemias” and “Diseases of the nervous system”, which are also linked to centrality and bursts in the network analyses. Of note, many of the genetic variants identified in this comparison are in linkage disequilibrium with variants associated with other human traits or diseases^46,47^, including smoking dependence^48^. This observation further reinforces the pleiotropic connection between the two diseases and others, and the possibility of identifying markers for estimating and/or preventing the risk of multimorbidity including those conditions. Prospective studies to address these questions may be warranted.

## Material and Methods

### Design, setting and study population

A cross-sectional study was conducted in Catalonia (Spain), a Mediterranean region with 7,434,632 inhabitants, 81% of whom live in urban municipalities (2010 census). The Spanish National Health Service (NHS) provides universal coverage, financed mainly by tax revenue. The Catalan Health Institute (CHI) manages primary health care teams (PHCTs) that serve 5,501,784 patients (274 PHCTs), or 74% of the population; other providers manage the remaining PHCTs. The CHIs Information System for the Development of Research in Primary Care (SIDIAP) contains the coded clinical information recorded in electronic health records by its 274 PHCTs since 2006. A subset of records meeting the highest quality criteria for clinical data (SIDIAP-Q) includes 40% of the SIDIAP population (1,833,125 individuals), attended by 1,365 general practitioners whose data recording scored highest in a validated comparison^25^. SIDIAP has been shown to be highly representative of the Catalan general population in terms of geography, age and gender distributions according to the official 2010 census. This study included individuals ≥19 years of age and assigned to a PHCT during the period of study (1^st^ January–31^st^ December 2010). The SIDIAP-Q study was approved by the Jordi Gol University Institute for Research Primary Healthcare (IDIAP) ethics committee and the GWAS by the Germans Trias i Pujol Health Sciences Research Institute (IGTP) ethics committee. Regarding SIDIAP and according to Spanish legislation about confidentiality and data protection (Organic Law 15/1999 of 13 December for the Protection of Personal Data), the data included in this database were always anonymized; thus, it was not necessary to ask for informed consent to the participants. All the participants in the GCAT GWAS provided written informed consent. These studies followed national and international regulations for research involving human subjects: Declaration of Helsinki Ethical Principles for Medical Research Involving Human Subjects and Good Research Practice principles and guidelines. The SIDAP-Q data are available upon request and ethics committee approval, and GCAT GWAS data have been deposited in the European Genome-phenome Archive.

### Coding and selection of diseases

Diseases are coded in SIDIAP using the ICD-10^27^. For this study, we selected all active diagnoses recorded in electronic health records as of December 31^st^ 2010, except for *R* (symptoms, signs, and abnormal clinical and laboratory findings, not elsewhere classified) and *Z* (factors influencing health status and contact with health services) codes. Non-active diagnoses, identified by the presence of an end date in the records, were excluded from the analysis. These diagnoses cover a broad list of acute diseases for which the system automatically assigns an end date (e.g., 60 days after the initial diagnosis). To facilitate management of the information, the diagnoses were extracted using the 263 blocks (disease categories) in the ICD-10 structure. These are homogeneous categories of very closely related specific diagnoses; for example, hypertensive diseases include “Essential (primary) hypertension, Hypertensive heart disease, Hypertensive renal disease, Hypertensive heart and renal disease, and Secondary hypertension”. From the 263 blocks, we excluded the *R* and *Z* codes, and 13 codes were not found in SIDIAP-Q, leaving 241 blocks suitable for analysis. To produce consistent and clinically interpretable networks based on binary disease associations, and to avoid inclusion of spurious relationships that could bias the results, we considered only diagnoses with ≥1% prevalence for each of the following age strata: 19–24, 25–29, 30–34, 35–39, 40–44, 45–49, 50–54, 55–59, 60–64, 65–69, 70–74, 75–79, 80–84, 85–89, ≥90 years, and for both genders. This minimum threshold of prevalence led to the analysis of 144 and 141 diseases in men and women, respectively. All patients with two or more coexisting diagnoses recorded on 31^st^ December 2010 were included.

### Chronic and non-chronic definition

Each diagnosis was classified using the O’Halloran criteria for chronic conditions in the International Classification for Primary Care-2 (CIAP-2)^58^. We included all 146 diagnoses considered as chronic diseases by these criteria: i) having a duration that has lasted, or is expected to last, at least six months; ii) having a pattern of recurrence or deterioration; iii) having a poor prognosis; or iv) producing consequences, or sequelae, that have a significant impact on quality of life. The diseases that did not meet these criteria were classified as non-chronic. The ICD-10 codes were mapped to identify chronic and non-chronic diseases. All results were described using these codes.

### Relative risk computation and trends

Categorical variables were summarized as frequencies (percentages); normally and non-normally distributed quantitative variables were summarized as means (standard deviations, SDs) and medians (interquartile ranges, IQRs), respectively. The relative risk (RR) was calculated to quantify the strength of disease associations (comorbid if RR > 1 or tending to be mutually exclusive if RR < 1) as previously proposed^15,28^. The ratio is that of the observed prevalence of patients diagnosed with both diseases to the expectation based on the product of the corresponding disease prevalences. The RR 95% confidence intervals (CIs) and *P* values were obtained using the methods of Katz^59^, and Altman and Bland^60^, respectively. Generalized additive models (GAMs)^61^ using cubic splines as the smoothing function were fitted to estimate RR sum distributions over age groups, for RR > 1 and RR < 1 associations, stratified by gender. The 95% CI of each distribution was obtained from the standard error of the fitted model. Join-point models (https://surveillance.cancer.gov/help/joinpoint) were used to investigate the trends of RR sum distributions across age groups. Statistical differences in slope were assessed using the annual percent change (APC) test^62^. To check the similarity of any pair of RR sum distributions tests for parallelism and coincidence^63^ were conducted. The cumulative distributions of disease associations across age groups were evaluated using the RR and Jaccard index (particularly the 1-Jaccard) estimates, and three similar approaches: 1) by computing the sum of the association estimates for each disease in each stratum and gender; 2) by computing the average of the estimates for each disease in each stratum and gender; and 3) by computing the sum of the estimates for each disease in each stratum and gender, but considering only diseases with ≥1% prevalence and dividing the sum by the number of strata in which a given disease appears. The correlation relative to the network centrality values was computed using, for each disease, the difference between the minimum and maximum of the cumulative estimate across age groups. The centrality values were normalized between 0 and 1, and the average value across all the networks was used for each disease.

### Network construction

For each age group and gender (i.e., stratum), a network was built with nodes corresponding to diagnoses matching the criteria detailed above, and edges corresponding to comorbidity if the corresponding RR was included in the top or bottom vigintile of the overall distribution of RRs in a given stratum. These percentiles corresponded to RRs < 0.8 or >1.6 across all strata. The SIDIAP-Q dataset linked the diagnoses in each stratum using the Jaccard index, *J*_*ij*_^26^. This index accounts for the similarity of two diagnoses *d*_*i*_ and *d*_*j*_, and takes values between 0 and 1. In parallel, the SIDIAP-Q dataset contained the frequency of the diagnoses, *N*_*i*_ and *N*_*j*_, and the population number *N* for each stratum. From these data, RRs were computed as follows:

$$R{R}_{ij}=\frac{[{J}_{ij}({N}_{i}+{N}_{j})/(1+{J}_{ij})]N}{{N}_{i}{N}_{j}}$$

With the criteria of considering diagnosis with prevalence greater than 1%, and discarding disease associations based on their RR percentiles (>5% and <95%), the networks contained between 73 and 111 diagnoses. The number of these diagnoses varied with age and gender, whereby more nodes were generally noted for women and for older age groups.

### Small-world-ness

In order to assess the small-world-ness characteristic of the observed morbidity networks, we used the method proposed by Humphries and Gurney^32^. The approach states that a small-world network fulfills the condition that ${L}_{G}\ge {L}_{{\rm{rand}}}$ and ${{\rm{C}}}_{G}^{{\Delta }}\gg {C}_{{\rm{rand}}}^{{\Delta }}$, where *L* is the average shortest path length of the network and *C*^*Δ*^ is the average clustering coefficient. The small-world-ness *S*^*Δ*^ is introduced as follows:

$${S}^{{\Delta }}=\frac{{C}_{G}^{{\Delta }}/{C}_{{\rm{rand}}}^{{\Delta }}}{{L}_{G}/{L}_{{\rm{rand}}}}$$

Therefore, *S*^*Δ*^ > 1 corresponds to a small-world network. In this study, we did not consider different weights for the network edges (i.e., all weights had a value of 1). The ${C}_{{\rm{rand}}}^{{\Delta }}$ and *L*_rand_ values were, for each network, the average of 1,000 *G*_*n*,*m*_ random model sample values. The outcomes were *S*^*Δ*^ > 1 for all observed networks and *S*^*Δ*^ > 2 for the strata younger than 80 years of age.

### Node centrality

The PageRank^35^ algorithm was used to compute node centrality in the networks. This algorithm assigns a weight to each node that ranks its importance among the global set of nodes of the network. A node that is related, either directly or through other nodes, to nodes with a high PageRank value receives a higher weight and is defined as more “central”. The PageRank can be considered a variant of the eigenvector and Katz centralities, and overcomes problems like the concentration of most of the centrality on a relatively small number of nodes^64^. The PageRank value of a node is defined recursively and determined by three main factors: the number of edges it receives and their weight; the number of edges of the neighbors; and the centrality of these neighbors. This ranking algorithm has a probabilistic interpretation using the so-called Google matrix *G*^65^. For an undirected positive edge weighted graph, *G* is defined as follows:

$$G=\alpha W{D}^{-1}+\frac{1-\alpha }{n}J$$

Therefore, *α* is the damping factor, *W* is the weighted adjacency matrix of the network, *D* is the diagonal degree matrix defined by *D*_*ij*_ = *∑*_*j*_ |*W*_*ij*_|, and *J* is the matrix of all ones. The matrix *G* is a left-stochastic Markov matrix —each column sums to one— and represents random walks in the network. The parameter (1 − *α*) is the probability of jumping randomly to any node in the Markov chain process without having to follow an edge between the nodes. The PageRank values are the entries of the dominant right eigenvector, which correspond to the steady-state of the Markov chain. The straightforward generalization of PageRank to signed weights, named signed spectral ranking^66^, raises a problem: *G* is no longer a stochastic matrix, so the probabilistic interpretation loses meaning. To resolve this limitation, we used a method that considers positive (*G*^+^) or negative (*G*^−^) weights^67^ to compute PageRank values for each sub-graph *PR*^+^ and *PR*^−^, respectively, thereby obtaining the final rank vector as *MPR* = *PR*^+^ − *PR*^−^, where *MPR* stands for the Modified PageRank. The damping parameter is usually assumed to be *α* ≈ 0.85 for technical and social networks. As there is no established guideline for setting this value, we used *α* = 0.5 to take into account the fact that nodes represent blocks of diseases. Different values of *α* might change the order of the ranking, but high-ranked nodes persist. The human disease gene network was built using DisGeNet curated gene-disease associations (version 5.0)^39^. The distance matrix between all vertices was computed and closeness centrality determined for each vertex as the inverse of the average distance to all other vertices. The eigenvector centrality was computed using the package NetworkX v2.1. All computations were performed using Python v2.7. Similar analyses were performed using the Agile Protein Interactomes DataServer (APID) level 2 dataset, which includes protein interactions proven by two or more experiments^40^.

### Community detection

It is assumed that a community (or clustering) division separates the nodes of the network into groups such that connections are stronger or more frequent within groups than between them. This study took a heuristic approach based on the maximization of modularity, a commonly used community quality measure. Modularity, *Q*, is a function representing the difference between the total edge weight in sets of the network under study and the total expected weight in the same sets from a random network generated by a given null model:

$$Q=\frac{1}{2m}\sum _{i}\,\sum _{j}\,({A}_{ij}-{P}_{ij}){\delta }_{{\sigma }_{i},{\sigma }_{j}}$$

where *m* is the number of edges in the network, *A*_*ij*_ is the (*i*, *j*) element of the adjacency matrix, *P*_*ij*_ is the null term, and ${\delta }_{{\sigma }_{i},{\sigma }_{j}}$ is the Kronecker *δ* between the communities of nodes *i* and *j*, that is *σ*_*i*_ and *σ*_*j*_, respectively. With the correct choice of the null model it is possible to incorporate specific features of the network structure. A standard choice is *P*_*ij*_ = *k*_*i*_*k*_*j*_/2 *m*, where *k*_*i*_ and *k*_*j*_ are the degrees of nodes *i* and *j*, respectively^68^. For a weighted signed network, the modularity function *Q* can also be defined using the appropriate null model. This model should take into account the so-called “resolution limit”: modularity optimization might fail to identify small communities. The resolution scale depends on the total size of the network and the interconnectedness of the communities. A possible solution to this problem is to scale the signed null model by introducing parameters γ^+^ and γ^−^. The former equation then becomes:

$$Q=\frac{1}{2{w}^{+}+2{w}^{-}}\,\sum _{i}\,\sum _{j}\,[{W}_{ij}-({\gamma }^{+}\frac{{w}_{i}^{+}{w}_{j}^{+}}{2{w}^{+}}-{\gamma }^{-}\frac{{w}_{i}^{-}{w}_{j}^{-}}{2{w}^{-}})]{\delta }_{{\sigma }_{i},{\sigma }_{j}}$$

where *W* is the signed weighted adjacency matrix of the network,

$$W=[{\tilde{RR}}_{ij}]\in {{\mathbb{R}}}^{n\times n}$$

with

$${\tilde{RR}}_{ij}=\{\begin{array}{cc}R{R}_{ij} & {\rm{if}}\,R{R}_{ij} > 1\\ -\,1/R{R}_{ij} & {\rm{if}}\,R{R}_{ij} < 1\end{array}$$

${w}_{i}^{+}$ and ${w}_{i}^{-}$ are signed generalized degrees from

$$\begin{array}{rcl}{w}_{i}^{+} & = & \sum _{j}\,{\rm{\max }}(0,{W}_{ij})\\ {w}_{i}^{-} & = & \sum _{j}\,{\rm{\max }}\,(0,-\,{W}_{ij})\\ {w}_{i} & = & {w}_{i}^{+}-{w}_{i}^{-}\end{array}$$

and the values of γ^+^ and γ^−^ determine the importance assigned to the null network. Increasing γ^+^ enables smaller communities to be detected. On the other hand, smaller groups of nodes can be detected by decreasing γ^−^. A method for estimating the best values of γ^+^ has recently been described^69^ and it is extended in this study to estimate γ^−^. The community configuration *σ* is obtained by maximizing *Q*. The number of possible community configurations in a network of *n* nodes is given by the Bell number, which grows exponentially with *n*. This is an NP-hard problem^70^, so heuristic algorithms are required. This study employed a method known as “spin glass community detection”^71^, an approach from statistical physics and based on the Potts model. In this model, each particle can be in one of several spin states, and the interactions between them determine which particles would prefer to have the same spin state. The analogy links particles with nodes, interactions with edges, and communities with the spin states. One aims to minimize the energy of the system, denoted by the Hamiltonian ${\mathcal H}$, in order to find the ground state. It is known that the ground state is the most stable configuration of the system, and hence a cohesive community structure. An extension of the spin glass method to signed weighted networks was implemented in python-igraph for use in this study. The Hamiltonian ${\mathcal H}$, which rewards internal positive and absent negative edges, and penalizes absent internal positive and internal negative edges^72^, is given as:

$$ {\mathcal H} =-\,\sum _{i}\,\sum _{j}\,[{W}_{ij}-({\gamma }^{+}\frac{{w}_{i}^{+}{w}_{j}^{+}}{2{w}^{+}}-{\gamma }^{-}\frac{{w}_{i}^{-}{w}_{j}^{-}}{2{w}^{-}})]\,{\delta }_{{\sigma }_{i},{\sigma }_{j}}$$

The Hamiltonian ${\mathcal H}$ and the modularity *Q* are related by

$$Q=-\,\frac{1}{2{w}^{+}+2{w}^{-}} {\mathcal H} $$

and, consequently, minimizing ${\mathcal H}$ implies maximizing *Q*. The algorithm implemented uses a classical simulated annealing method^73^ to solve the combinatorial problem. This technique can find a good solution, even when there is some noise in the data. Using a probabilistic process, it approximates the global optimum of the given function.

### Community independence and roots

To qualitatively rank the degree of independence of a community we used an adaptation of the degree centrality that relies on ${\tilde{R}}_{ij}$ to reward a community for its negative interactions with the other communities and to penalize it for positive interactions. The method is detailed by the following equation, where higher values imply greater independence:

$$I({\sigma }_{k})=\frac{1}{{n}_{k}}\,\sum _{i}\,\sum _{j}\,-\,{\rm{sign}}\,({W}_{ij})\,|{W}_{ij}|{\delta }_{{\sigma }_{k},{\sigma }_{i}}(1-{\delta }_{{\sigma }_{k},{\sigma }_{j}})$$

Therefore, *I*(*σ*_*k*_) accounts for the independence of community *σ*_*k*_, *n*_*k*_ corresponds to the number of nodes in the community, *W*_*ij*_ is the signed weighted adjacency matrix, and ${\delta }_{{\sigma }_{k},{\sigma }_{i}}$ is the Kronecker *δ* of communities *σ*_*k*_ and *σ*_*i*_. A root was defined by the detection of at least two nodes in a given community across a minimum of two consecutive strata. The three highest ranked/most independent (as defined above) communities in each stratum were analyzed.

### Coherence of disease pairs

The biological coherence of the morbidity associations was assessed by analyzing the shortest path distance in a high-quality network of molecular interactions^15^ between genes and/or proteins (genes/proteins) assigned to diseases, then comparing the results with those of random genes/protein pairs. Causal genes/proteins were assigned on the basis of phenotype-genetic annotations extracted from the OMIM^74^ database. The OMIM annotations were linked to ICD-10 diagnoses using Metathesaurus included in the Unified Medical Language System (UMLS) version 2015AB^75^. All the diseases with at least one causal gene/protein were included in the analysis; 111 diseases had at least one causal gene/protein, of which 104 had at least one causal gene/protein represented in the molecular network. For each disease pair present in the observed male or female morbidity networks, we computed the average shortest path between their causal genes/proteins (e.g., shortest path between *gene*/*protein*_*i*_ and *gene*/*protein*_*j*_ corresponding to the associated diseases *i* and *j*, respectively) and compared the result with the average of 1,000 gene/protein pairs for which one of the members was randomly chosen and the other was a defined casual gene/protein. OMIM diseases frequently have more than one causal gene/protein and so we computed the average shortest path between the assigned genes/proteins. The morbidity networks included 1,051 and 1,031 disease associations by RRs > 1.6, and 239 and 206 disease associations by RRs < 0.8 in men and women, respectively, with OMIM-assigned causal genes/proteins. Thus, for each of these associations, we computed the average shortest path and compared the result with that of 1,000 random genes/proteins, thereby obtaining empirical *P* values. The analysis was performed using the complete interactome dataset compiled by Menche *et al*.^15^ or a subset corresponding to interactions with evidence from the literature and binary protein-protein assays.

### Degree leaps

To find leaps in the degree (*k*) of nodes through time, we constructed connectivity trajectories. For each diagnosis, we differentiated between edges corresponding to RRs > 1.6 or RRs < 0.8. Given a diagnosis or node, its connectivity trajectory for RRs > 1.6 was built as follows: for each age stratum, the number of connected edges with RRs > 1.6 was counted and the connectivity trajectory was the result of the number of edges across strata. Therefore, the leaps were defined based on the difference between the maximum and minimum *k* values of each node. Nodes with no change in their *k* across the age groups where they appear and nodes with spurious changes (a single change with no continuation in subsequent strata) were not considered in this analysis. To assess the observed distributions of leaps of disease connectivity across age in men and women, we generated random undirected networks that preserved the original node degree distributions and connectedness. The *latmio_und* function (Brain Connectivity MATLAB Toolbox; https://sites.google.com/site/bctnet/) was used for this analysis. Randomization was carried out using the “rewiring” parameter corresponding to the exact number of nodes in each observed network in the analysis. Thus, 1,000 random networks for each age group and gender were generated and combined (consecutively, one random network from each age group/gender) to obtain 1,000 random distributions of disease connectivity leaps, which were compared with the observed values.

### Gene set overlap, gene expression and pathway enrichment

The overlaps with the diabetic neuropathy and undernutrition gene expression signatures were computed using 2 × 2 contingency tables and the χ^2^ test with Yate’s correction, considering an approximate total of 18,500 annotated human genes (the actual number varying by study). Pre-processed and normalized RNAseq data of normal breast tissue and primary breast tumors were taken from The Cancer Genome Atlas (TCGA) repository (Data Access Committee project #11689). A paired *t*-test was applied to detect differentially expressed genes between the normal tissue and tumors, and in the overlap analysis we only considered the genes corresponding to a false discovery rate (FDR) of <1%. The Reactome enrichment tool^76^ was used with standard parameters to detect significant pathways with a FDR < 5%. The expression signature scores were computed using the ssGSEA algorithm^77^ with standard parameters and using all genes included in each signature. The linear correlation analysis between the signature scores and age at diagnosis was adjusted by tumor stage. The association between pleiotropy and smoking cessation/dependence gene targets was based on PubMed enrichment analysis using the DAVID tool^78^.

### GWAS analyses

The GCAT project includes a large prospective cohort from the Catalan general population with ages ranging between 40 and 65 years, baseline epidemiological characterization, and electronic health record-linked data^45,79^. For this study, we used baseline data at recruitment (2014–2016) for a subset of subjects. The participants (*n* = 5,459; GCATcore) were genotyped using the Expanded Multi-Ethnic Genotyping Array (MEGAEX) (Illumina). Genotyping was performed at the Genomics Unit IMPPC-IGTP. Extended quality control protocol is available at www.genomesforlife.com/GCATCoreAnalysis. After filtering, 4,988 participants and 1,652,023 genetic variants were included in the analysis. Sexual and mitochondrial chromosomes were discarded as well as autosomal chromosome variants with minor allele frequency (MAF) < 0.01 and AT-CG sites. Imputation used 665,592 (40%) variants and was performed using Shape-IT^80^ and IMPUTE2^81^ and four reference panels: 1000 Genomes, Genome of the Netherlands, UK10K, and Haplotype Reference Consortium. All variants with imputation correlations <0.7 were removed. The best score was used for those variants present in more than one reference panel. Variant dosage from IMPUTE2 was transformed to binary PLINK^82^ format by using the “hard-call-threshold 0.1” flag. The final core set was produced by approximately 15 million variants with MAF > 0.001 and 9.5 million variants with MAF > 0.01. Imputation was done at the Barcelona Supercomputing Center (BSC). Clinical conditions were defined from a self-reported questionnaire at baseline; 159 conditions were identified, occurring in 1 to 985 cases, 17 of these were collected by direct query, and some were identified from the open text field query. All reports were curated and mapped to ICD-10 codes. The diagnoses with more than 200 cases included: allergies, arterial hypertension, asthma, depression, dermatitis, hyperlipidemia, migraine, rhinitis, and type II diabetes. The analysis for association signals influencing these diagnoses comprised two consecutive steps: an individual GWAS analysis for each ICD-10-based disease and then a pairwise analysis to detect shared associations. The GWAS summary statistics, with quality control protocols and data are available at the GCAT website, and the raw data have been deposited at the European Genome-phenome Archive^83^ (access is regulated by GCAT Data Access Committee applications). The analyses were performed using the score test and saddlepoint approximation in the SPAtest R package^84^. This method accounts for unbalanced case-control designs, as was the case in our study. The 20 first dimensions of the principal component analysis of population substructure, gender and age data were included as covariates in all analyses. The variants with a nominal value of *P* < 0.05 in any of the considered single disease analyses were selected for pairwise analysis using the GWAS-pw tool^46^. This tool provides Bayes factor calculations and identifies variants that are shared in pairs of traits. Statistical power is assessed using a log Bayes factor >6^85^ and posterior probability >0.7. In addition, the level of significance of each comparison was inferred empirically from 100 random GWAS-pw tests, based on 10 independent simulated datasets of each pair of conditions. For the comparison between “Nutritional anemias” and “Diseases of the nervous system”, to determine the random frequency of variants in linkage disequilibrium (*D*’ > 0.99 and *P* < 0.05 in the Iberian population of Spain) with GWAS signals of any human trait, we generated 100 random sets of 31 variants (genome version hg19 and minor allele frequency > 0.01) and subsequently computed their degree of linkage disequilibrium in the same 1,000 Genomes population against variants from the GWAS catalog (v1.0.1, 2018-02-28)^86^ over a range of ±100 kb.

## Electronic supplementary material

Supplementary Figures S1-S6

Table S1

Table S2

Table S3

Table S4

Table S5

Table S6

Table S7

Table S8
