# Supplementary material for: Disease networks identify specific conditions and pleiotropy influencing multimorbidity in the general population
Source: Sci Rep. 2018 Oct 29;8:15970. doi: 10.1038/s41598-018-34361-3 (PMC6206057; doi:10.1038/s41598-018-34361-3)

Disease networks identify specific conditions and pleiotropy influencing multimorbidity in the general population

A. Amell, A. Roso-Llorach, L. Palomero, D. Cuadras, I. Galván-Femenía, J. Serra-Musach, F. Comellas, R. de Cid, M.A. Pujana & C. Violán

Supplementary Figures

Figure S1. Data and RR thresholds. (**a**) Description of the study population and SIDIAP-Q dataset. (**b**) Graph depicting the top and bottom vigintiles of RR thresholds, by gender and stratum.

Figure S2. Networks and small-word-ness. Small-world-ness property; all networks had values of $S^{\Delta} > 1$, which defines the threshold for the property.

Figure S3. Central nodes attack. Plots showing the number of network components resulting from eliminating central nodes in each disease network by age and gender (cross marks), or equivalent random sets of diseases (box plots). Observed higher numbers of network components relative to random attacks ($P_{\mathrm{empirical}} < 0.005$) are indicated by colored age groups.

Figure S4. Cumulative risks. Age-based trajectories of the RR sums for all diseases studied. Left and right panels show diseases linked by RRs > 1 and RRs < 1, respectively; top and bottom panels show results for men and women, respectively.

Figure S5. Average cumulative RR trend distributions. Trends for disease sets classified as central, community roots, or with large degree leaps, and using the average RR cumulative risks. The results are similar to those depicted in Fig. 4; that is, central diseases and those with large degree leaps show greater cumulative risks relative to the rest of diseases. Conversely, particularly in women, diseases classified as community roots have lower cumulative risks. Central and root diseases are marked in the insets, and the arrows indicate differences in each setting.

Figure S6. Centrality in the APID interactome. Graphs showing the distributions of closeness and eigenvector centrality measures for different types of causal genes as indicated in the insets. The results correspond to the analysis of APID level 2 data, and are shown for men and women disease sets derived from the SIDIAP-Q networks study. The Wilcoxon test *P* values of the comparisons of distributions are shown.

Figure S1

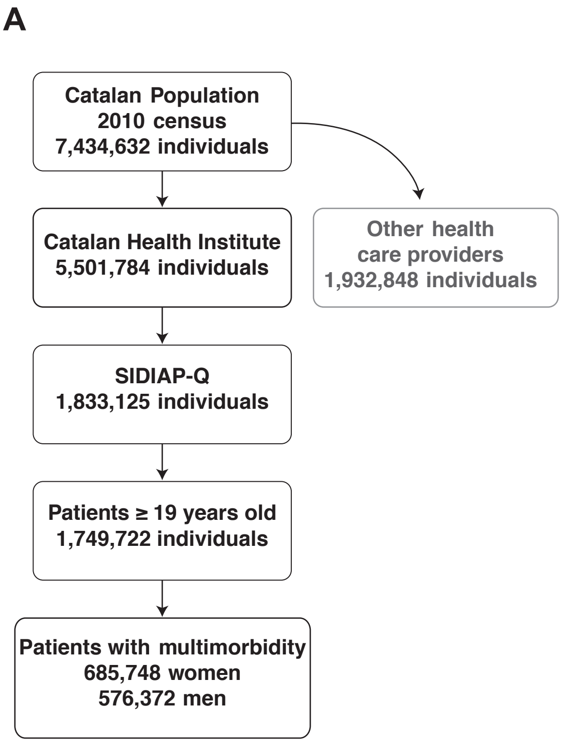

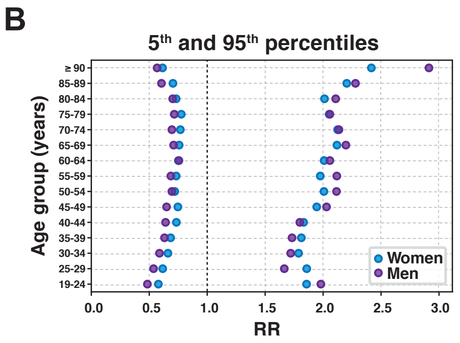

Figure S2

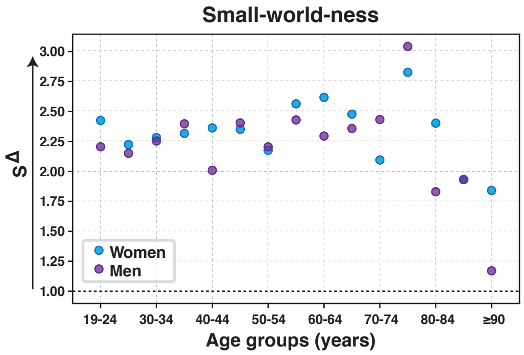

Figure S3

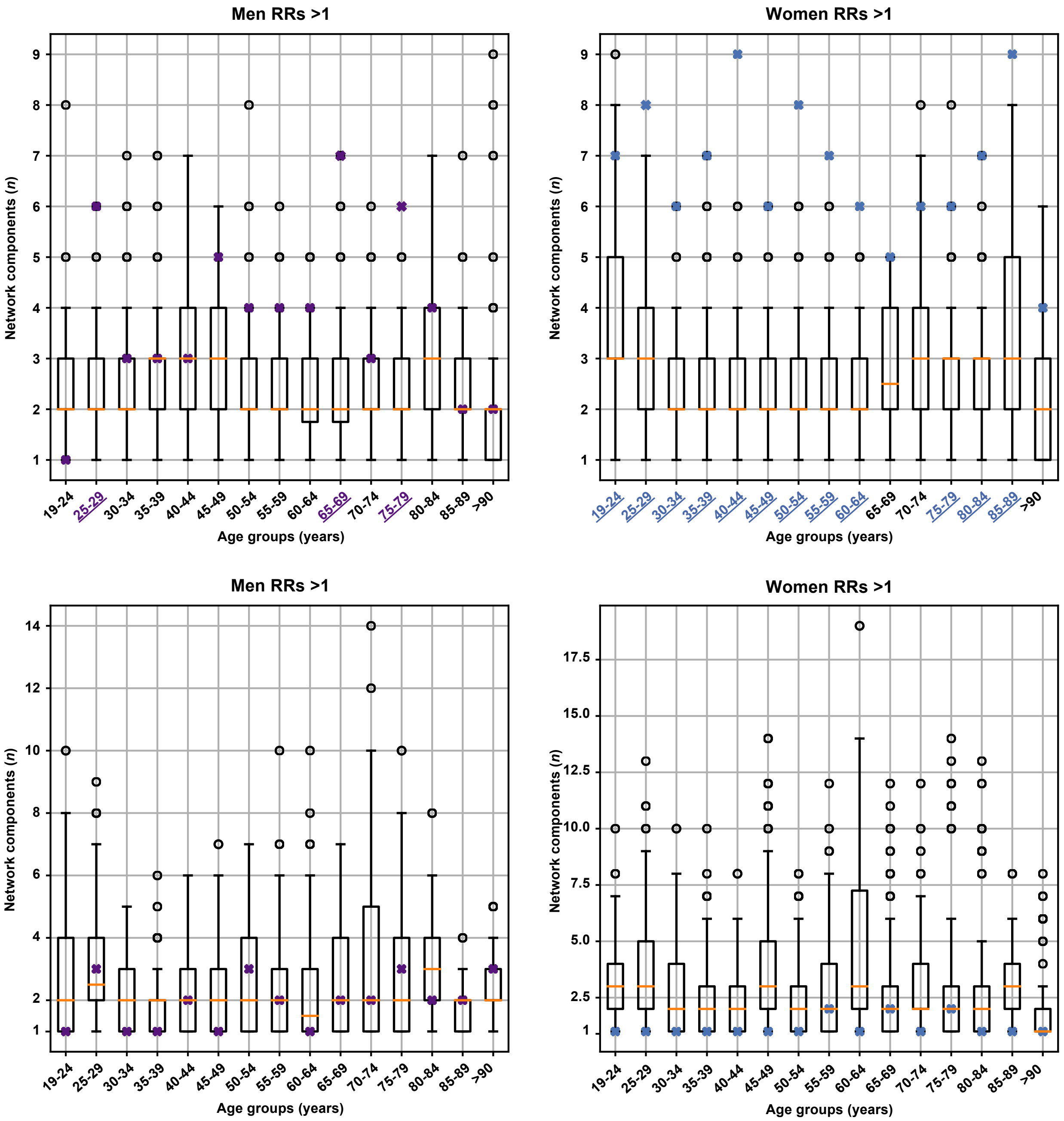

Figure S4

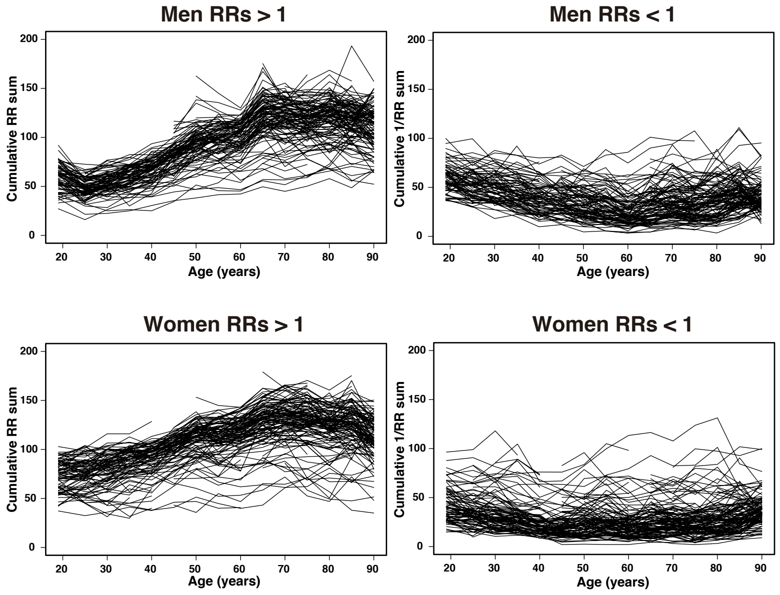

Figure S5

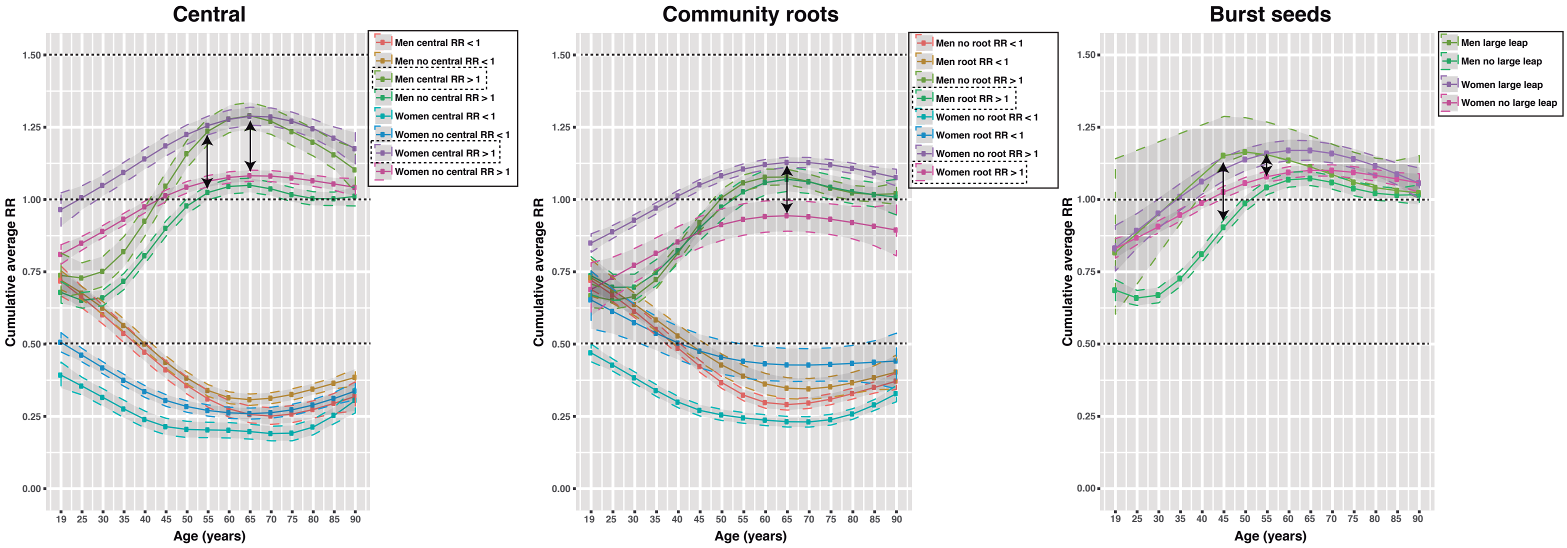

Figure S6

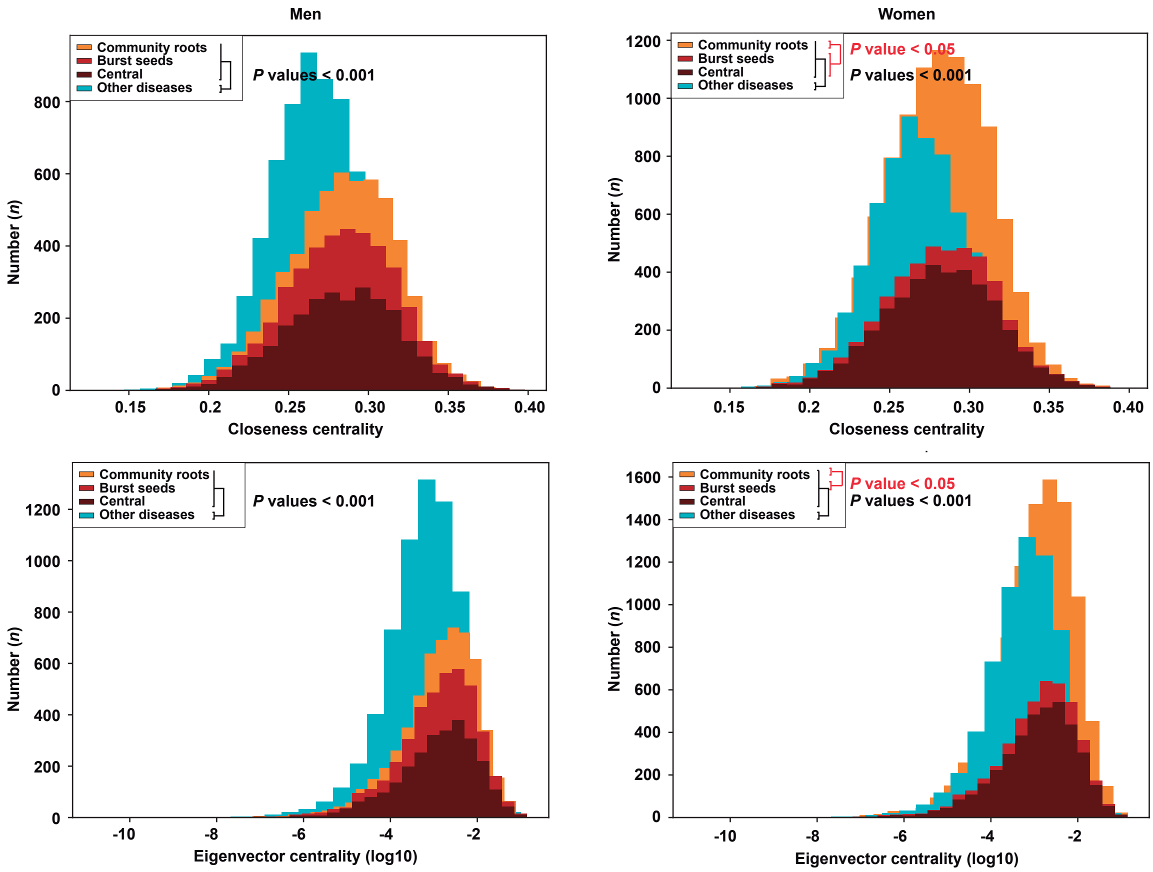

Supplement: Supplementary file 1 — Supplementary Figures S1-S6 [file 41598_2018_34361_MOESM1_ESM.pdf]
